# microRNA-145 promotes differentiation in human urothelial carcinoma through down-regulation of syndecan-1

**DOI:** 10.1186/s12885-015-1846-0

**Published:** 2015-10-29

**Authors:** Tomomi Fujii, Keiji Shimada, Yoshihiro Tatsumi, Kinta Hatakeyama, Chiho Obayashi, Kiyohide Fujimoto, Noboru Konishi

**Affiliations:** 1 Department of Pathology, Nara Medical University School of Medicine, 840 Shijo-cho, Kashihara, Nara 634-8521 Japan; 2 Department of Urology, Nara Medical University School of Medicine, Nara, Japan; 3 Department of Diagnostic Pathology, Nara Medical University School of Medicine, Nara, Japan

**Keywords:** Urinary bladder cancer, MicroRNA, MiR-145, Syndecan-1

## Abstract

**Background:**

A new molecular marker of carcinoma in the urinary bladder is needed as a diagnostic tool or as a therapeutic target. Potential markers include microRNAs (miRNAs), which are short, low molecular weight RNAs 19–24 nt long that regulate genes associated with cell proliferation, differentiation, and development in various cancers. In this study, we investigated the molecular mechanisms by which miR-145 promotes survival of urothelial carcinoma cells and differentiation into multiple lineages. We found miR-145 to regulate expression of syndecan-1, a heparin sulfate proteoglycan.

**Methods:**

Cell proliferation in the human urothelial carcinoma cell lines T24 and KU7 was assessed by MTS assay. Cellular senescence and apoptosis were measured by senescence-associated β-galactosidase (SA-β-gal) and TUNEL assay, respectively. Quantitative RT-PCR was used to measure mRNA expression of various genes, including syndecan-1, stem cell factors, and markers of differentiation into squamous, glandular, or neuroendocrine cells.

**Results:**

Overexpression of miR-145 induced cell senescence, and thus significantly inhibited cell proliferation in T24 and KU7 cells. Syndecan-1 expression diminished, whereas stem cell markers such as SOX2, NANOG, OCT4, and E2F3 increased. miR-145 also up-regulated markers of differentiation into squamous (p63, TP63, and CK5), glandular (MUC-1, MUC-2, and MUC-5 AC), and neuroendocrine cells (NSE and UCHL-1). Finally, expression of miR-145 was down-regulated in high-grade urothelial carcinomas, but not in low-grade tumors.

**Conclusions:**

Results indicate that miR-145 suppresses syndecan-1 and, by this mechanism, up-regulates stem cell factors and induces cell senescence and differentiation. We propose that miR-145 may confer stem cell-like properties on urothelial carcinoma cells and thus facilitate differentiation into multiple cell types.

**Electronic supplementary material:**

The online version of this article (doi:10.1186/s12885-015-1846-0) contains supplementary material, which is available to authorized users.

## Background

Urinary bladder cancer is a common malignant tumor with high mortality. Thus, well characterized biomarkers are needed to screen, diagnose and manage bladder cancer. Over the years, a number of studies have examined molecular mechanisms of urothelial carcinoma progression, as well as diagnostic tools and therapeutic strategies [1, 2]. However, useful diagnostic markers that predict clinical outcome have not been found. Potential new biomarkers include microRNAs (miRNAs), which are small non-coding RNAs that regulate gene expression by suppressing protein translation or by promoting degradation of messenger RNA [3]. Therefore, investigation of miRNAs and their targets could provide both novel therapeutic targets as well as diagnostic markers of the development, progression, differentiation, and metastatic potential of malignant tumors. Indeed, some miRNAs are abundantly expressed and act as tumor suppressors. MiRNAs such as miR-1, 100, 101, 133, and 192 are down-regulated in several cancers and promote cell proliferation, migration and invasion [4–7].

Syndecan-1 is expressed in a majority of epithelial and nonepithelial neoplasms, and is involved in cell growth, adhesion, migration, epithelial morphogenesis, and angiogenesis [8–10]. We previously reported that this proteoglycan transforms androgen-dependent prostate cancer cells into androgen-independent tumors and facilitates progression [11, 12]. Moreover, syndecan-1 and the miRNAs miR-126 and, 149 regulate cell proliferation through down-regulation of several stem cell factors such as SOX2, NANOG, and OCT4 [13]. Finally, syndecan-1 was detected in plasmacytoid urothelial carcinoma, an aggressive tumor [14–18], demonstrating that the protein is closely associated with urothelial carcinoma progression [19].

In this study, we demonstrate that miR-145 in bladder cancer cells down-regulates syndecan-1 expression, inhibits cell proliferation by inducing senescence, and promotes differentiation into glandular, squamous, and neuroendocrine cells. Moreover, miR-145 and syndecan-1 were found to be up-regulated in low-grade urothelial carcinoma, but not in high-grade tumors.

## Methods

### Cell lines

The human urothelial carcinoma cell lines, T24 was purchased from American Type Culture Collection (Manassas, VA), and KU7 was derived from human papillary bladder cancer [20]. T24 and KU7 were cultured in RPMI1640 media supplemented with 10 % fetal bovine serum and 50 units/mL penicillin-streptomycin at 37 °C in 5 % CO_2_.

### Transfection

T24 or KU7 were seeded at a density of 1x10^4^ cells/well and transfected for 72 h with Anti-miR™ miRNA Inhibitor, Pre-miR™ miRNA Precursor (hsa-miR-145, Life Technologies), or 100 ng/L siRNA against syndecan-1. Transfection was performed in Lipofectamine RNAiMAX (Life Technologies) according to the manufacturer’s protocol. The sequence of the syndecan-1 siRNA was 5′-TCCGACTGCTTTGGACCTAAA-3′.

### Quantitative RT-PCR

Total RNA, including miRNA, was extracted from cells using the miRNeasy Mini kit (QIAGEN). First-strand cDNA was synthesized from 1 μg total RNA using PrimeScript RT Master Mix (Perfect Real Time, TaKaRa), TaqMan MicroRNA Reverse Transcription Kit (Applied Biosystems) and TaqMan MicroRNA Assays (Applied Biosystems), respectively. real-time PCR was performed using SYBR Premix Ex Taq II (TliRNaseH Plus, TaKaRa) and TaqMan Universal PCR Master Mix II (Applied Biosystems), respectively. Templates were initially denatured at 95 °C for 30 s, and targets were amplified over 35–45 PCR cycles at 55-63 °C (Table 1).

**Table 1:** The sequence list of PCR primers

Syndecan-1	sense 5′-GGCTGTAGTCCTGCCAGAAG-3′
	antisense 5′-GTTGAGGCCTGATGAGTGGT-3′
p63	sense 5′-GAAAGCAGCAAGTTTCGGAC-3′
	antisense 5′-TTTCATAAGTCTCACGGCCC-3
TP63	sense 5′-CATCCATCAAGAAACGAAGATCCC-3′
	antisense 5′-AATTGTGTGCTGAGGAAGGTACTG-3
CK5	sense 5′-TCTCGCCAGTCAAGTGTGTC-3′
	antisense 5′-ATAGCCACCCACTCCACAAG-3
MUC-1	sense 5′-TCCAATATTAAGTTCAGGCCAGGA-3′
	antisense 5′-CACATCACTCACGCTGACGT-3
MUC-2	sense 5′-TGAAGACCTGCGGCTGTGT-3′
	antisense 5′-CAGTCGAACTCGAAGTGCTCC-3
MUC-5 AC	sense 5′-GGACAGCTCTTCCATGTACTCG-3′
	antisense 5′-CAGGGTCACATTCCTCAGCGA-3
NSE	sense 5′-CTTAGGCAAAGGTGTCCTGA-3′
	antisense 5′-TCCAGTTTCTCTTGCTCCAC-3
UCHL-1	sense 5′-CTCTATGAACTTGATGGACGAATGC-3′
	antisense 5′-GCGGACTTCTCCTTGCTCAC-3
SOX2	sense 5′-GACCAGCTCGCAGACCTACAT-3′
	antisense 5′-ATGGAGCCAAGAGCCATGC-3′
NANOG	sense 5′-AACCTCAGCTACAAACAGGTGAAG-3′
	antisense 5′-CTGCGTCACACCATTGCTATTCT-3′
4-Oct	sense 5′-TTCCCCCTGTCTCCGTCAC-3′
	antisense 5′-AGAACTTAATCCCAAAAACCCTGG-3′
E2F3	sense 5′-TGCCTGACTCAATAGAGAGCCTAC-3′
	antisense 5′-GTCTTTGGAAGCGGGTTTAGGG-3
CD44	sense 5′-AAGGTGGAGCAAACACAACC-3′
	antisense 5′-AGCTTTTTCTTCTGCCCACA-3
p53	sense 5′-TCTGAGTCAGGCCCTTCTGT-3′
	antisense 5′-GTTCCGAGAGCTGAATGAGG-3
E-cadherin	sense 5′-CAGCGTGTGTGACTGTGAAGG-3′
	antisense 5′-CAGCAAGAGCAGCAGAATCAGAA-3
GAPDH	sense 5′-ACCCACTCCTCCACCTTTGAC-3′
	antisense 5′-GCCAAATTCGTTGTCATACCAGGA-3

### Cell proliferation

CellTiter 96 AQueous One Solution Cell Proliferation Assay (Promega) was used for MTS assay as previously described [11] to measure cell proliferation. Data were collected from triplicate experiments.

### Senescence-associated β-galactosidase (SA-β-gal) assay and TdT-mediated dUTP nick end labeling (TUNEL) assay

SA-β-gal was measured using Senescence Detection Kit (BioVision, CA) in T24 cells transfected for 72 h with miR-145 precursor. The assay was performed according to the kit manual. Similarly, T24 cells transfected with miR-145 precursor for 72 h were harvested and stained with ApopTag Plus Peroxidase *In Situ* Apoptosis Detection Kit (Millipore Corporation, CA).

### Tissue samples

We examined fifteen trans-urethral resection of bladder tumor specimens without undergoing chemotherapy or Bacillus Calmette-Guerin treatment (age: 51–84 years, grade: low grade 5 cases; high grade 10 cases). The present study received ethics committee approval of Nara Medical University (NMU900). The informed consent was obtained from all patients. All tissue samples were fixed in 10 % formalin for 48 h and processed through graded alcohols to paraffin. Paraffin blocks were sectioned at 3-μm intervals and stained with hematoxylin and eosin (HE) for histological diagnosis. For each HE stained sample, corresponding sections, to include cancer foci of interest, were cut at 8-μm intervals for extraction of total RNA. Tumor stage and grade were noted at the time of diagnosis by two independent urological pathologists (KS and NK) (Fig. 4a). Total RNA, including miRNA, was purified from paraffin-embedded tissue sections using miRNeasy FFPE kit (QIAGEN). First-strand cDNA was synthesized using TaqMan MicroRNA Reverse Transcription Kit (Applied Biosystems), and real-time PCR was performed using TaqMan MicroRNA Assays and TaqMan Universal PCR Master Mix II (Applied Biosystems) to amplify miR-145 and RNU6B.

Informed consent was obtained from patients as appropriate before specimens were collected. The study was approved by the ethics committee at Nara Medical University.

### Statistical analysis

Differences in measures of continuous variables were analyzed using ANOVA or the nonparametric Mann–Whitney and Kruskal-Wallis tests. Results were analyzed using one-way ANOVA and Turkey’s post hoc test. Two-tailed Student’s *t* test was used to compare two data points. Results with *p* < 0.05 were considered significant.

## Results

### miR-145 suppresses cell proliferation in bladder cancer cells

**Fig. 1:**
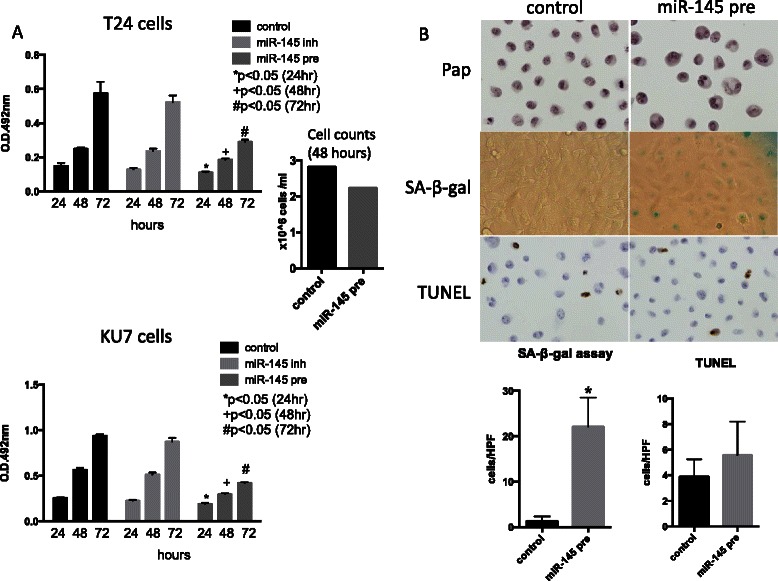
**a** MTS assay in T24 and KU7 cells. Cell proliferation was suppressed by transient transfection of miR-145 precursor. (inh:inhibitor, pre:precursor) The cell counts was also lower in miR-145 precursor transfected T24 cells (48 h). **b** Papanicolaou stain (upper panel), SA-β-gal (middle panel) and TUNEL (lower panel) assay. Papanicolaou stain of T24 cells showed slightly enlarged by transient transfection of miR-145 precursor in T24 cells. Senescent cells with SA-β-galactosidase activity was significantly induced, on the other hand, apoptotic cells were not increased by miR-145 overexpression (*p < 0.05)

We first examined whether miR-145 regulates cell proliferation in urothelial carcinoma cells by transfecting the miR-145 precursor into T24 and KU7 cells. Figure 1a shows that miR-145 overexpression significantly suppressed cell proliferation. To evaluate the mechanisms underlying this suppression, SA-β-gal and TUNEL assays were performed. We found senescence to be induced in overexpressing T24 cells, although the number of apoptotic cells was not significantly increased (Fig. 1b). Taken together, the data demonstrate that miR-145 regulates cell proliferation in urothelial carcinoma cells by inducing senescence, but not apoptosis.

### Expression of differentiation markers

**Fig. 2:**
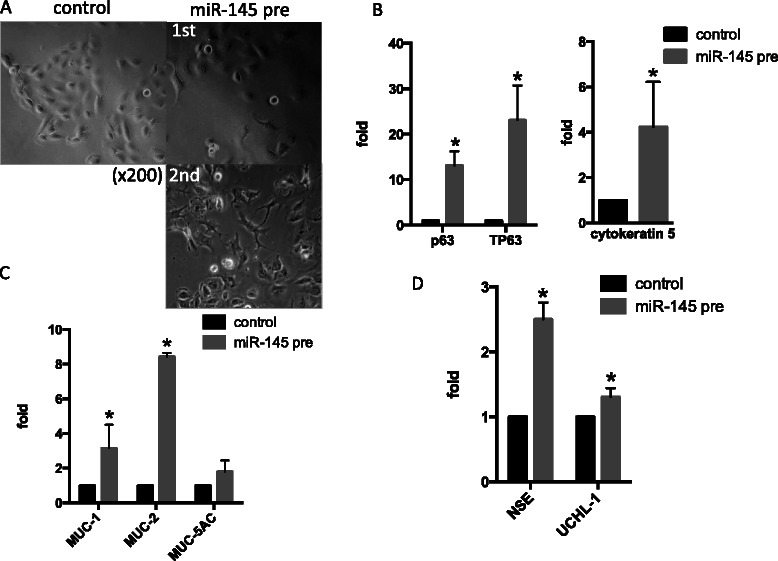
**a** Morphological comparison of miR-145 precursor transfected T24 cells cultured under adherent conditions. **b,c,d** mRNA expression of squamous markers (p63, TP63 and cytokeratin 5 : **b**, glandular markers (MUC-1, MUC-2 and MUC-5 AC : **c** and neuroendocrine markers (NSE and UCHL-1 : **d** was increased under conditions of miR-145 overexpression in T24 cells. Y-axis in **b,c** and **d** was indicative of relative mRNA expression compared with control. All mRNAs data were normalized by GAPDH. (*p < 0.05, miR-145 pre; miR-145 precursor)

miR-145 transfection slightly changed the morphological characteristics of T24 and KU7 cells. Therefore, cells were additionally transfected 48 and 72 h after initial transfection. Figure 2a shows elongated axis feet in transfected T24 cells. Cytoplasmic expansion is clearly observed in samples stained with Papanicolaou stain (Fig. 1b, upper panel). In addition, miR-145 transfection increased mRNA expression of some differentiation markers, including p63, TP63, and cytokeratin 5, which are markers of squamous cells, as well as MUC-1, MUC-2, and MUC-5 AC, which are markers of glandular cells. Neuroendocrine markers; neuron-specific enolase (NSE) and ubiquitin carboxyl-terminal esterase L1 (UCHL-1) were also induced (Fig. 2 b-d, Additional file 1: Figure S1A).

### miR-145 significantly induces NANOG, SOX2, OCT4, and E2F3

**Fig. 3:**
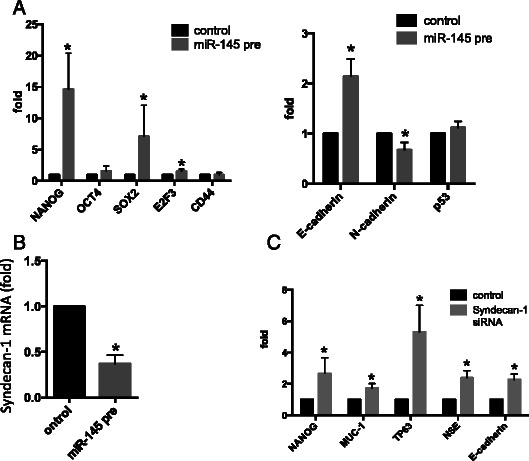
**a** mRNA expression of stem cell markers (SOX2, NANOG, Oct4, and E2F3) was increased but not CD44 under conditions of overexpression of miR-145 in T24 cells (Left panel). E-cadherin expression was increased, but not p53 under transfection of miR-145 precursor (Right panel). **b** Syndecan-1 mRNA expression was suppressed by transfection of miR-145 precursor. **c** NANOG, MUC-1, TP63, NSE amd E-cadherin increased in T24 cells following transient transfections of syndecan-1 siRNA. Y-axis in A, B and C was indicative of relative mRNA expression compared with control. All mRNAs data were normalized by GAPDH. (*p < 0.05, miR-145 pre; miR-145 precursor)

Morphological changes and induction of several differentiation markers indicate that urothelial carcinoma cells overexpressing miR-145 differentiate into various lineages. Accordingly, we found that stem cell markers such as NANOG, OCT4, SOX2, and E2F3 were strongly induced, but not CD44 (Fig. 3a, Additional file 1: Figure S1B). miR-145 also induced E-cadherin, but not p53 (Fig. 3a, Additional file 1: Figure S1C). As reduced E-cadherin expression is associated with high-grade, invasive carcinomas, and correlates with poor prognosis [21, 22], the data indicate that miR-145 facilitates differentiation into various cell types, but not invasion.

### miR-145 significantly decreases syndecan-1 expression

Overexpression of miR-145 significantly suppressed syndecan-1 mRNA (Fig. 3b, Additional file 1: Figure S1D). Similarly, silencing of syndecan-1 by siRNA up-regulated some stem cell and differentiation markers (Fig. 3c). Taken together, the data suggest that miR-145 regulates cell proliferation and differentiation in urothelial carcinoma cells by down-regulating syndecan-1 while up-regulating some stem cell markers.

### Expression of miR-145 in clinical bladder cancer tissues

**Fig. 4:**
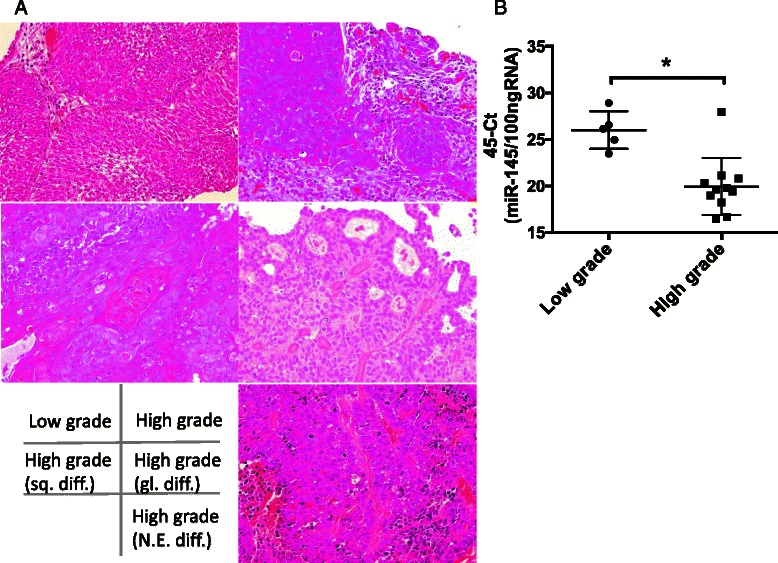
Expression of miR-145 in urothelial carcinoma tissues. **a** Urothelial carcinoma of the bladder tissues. Urothelial carcinomas were histologically classified in to low grade and high grade (sq. diff.: squamous differentiation, gl. diff. :glandular differentiation, N.E. diff.: neuroendocrine differentiation) (Hematoxylin-eosin staining). **b** qRT-PCR analysis of the urothelial carcinoma tissues showed that miR-145 highly expressed in low grade than high grade carcinomas. (*p < 0.05, low grade v.s. high grade)

*In vitro* results imply that miR-145 is suppressive, and impedes progression of urothelial carcinoma cells. Therefore, miR-145 might be a novel marker to accurately detect carcinoma cells in surgical tissue specimens. Urothelial carcinoma was histologically classified into low grade, high grade and high grade with squamous, glandular or neuroendocrine differentiation (Fig. 4a). Analysis of TUR tissue specimens clearly shows that expression of miR-145 is statistically lower in high-grade tumors than in low-grade, non-invasive, or superficially invasive tumors (Fig. 4b). These results suggest that miR-145 can be used as a novel molecular marker for histological diagnosis of bladder cancer.

## Discussion

In this study, we demonstrate for the first time that miR-145 in bladder cancer cells suppresses syndecan-1 and thereby regulates cell proliferation and expression of some markers of differentiation into squamous, glandular, and neuroendocrine cells. In addition, miR-145 induces expression of stem cell markers such as SOX2, OCT4, NANOG, and E2F3. Furthermore, the ability of miRNAs to reprogram cancer cells into cells with stem cell-like properties and low malignancy potential has been previously demonstrated in colon cancer [23], therefore miRNAs could regulate the fate of cancer cells. These results are in line with our recently published study, in which we demonstrate that syndecan-1, miR-126, and miR-149 modulate cell senescence by controlling expression of SOX2, NANOG, and OCT4 [13].

Differentiation into squamous or glandular cells is a hallmark of advanced-stage, invasive urothelial carcinomas with poor prognosis [24–26]. An isoform of p63, a marker of squamous cells, transactivates p53 target genes and induces cell cycle arrest and apoptosis [25, 26]. Up-regulation of MUC-1, a marker of glandular cells, promotes cell proliferation and invasion through epithelial-to-mesenchymal transition in some late-stage carcinomas including urothelial cancer [27–30]. UCHL-1 is overexpressed in pancreatic endocrine tumors, as well as in colorectal and lung cancers. This enzyme is a neuroendocrine marker that recognizes and hydrolyzes a peptide bond at the C-terminus of ubiquitin, and remodels synapses by controlling ubiquitin homeostasis [31–33]. In the current study, miR-145 provided to suppress cell proliferation and reverse urothelial carcinomas to the pluripotent cells with stem cell features through induction of senescence, then prompted differentiation to multiple lineages such as squamous, glandular and neuroendocrine cells. Taken together, miR-145 might contribute to the early step of divergent differentiation for invasive urothelial carcinomas.

miR-145 appears to be a tumor suppressor, the expression of which is reduced in various carcinomas including colon, lung, prostate, and breast cancer [34–37]. A number of recent studies have focused on the role of miR-145 in multiple cellular pathways underlying carcinogenesis. For example, miR-145 has been found to inhibit cancer cell growth, invasion, and metastasis by suppressing EGFR and NUDT1 in lung adenocarcinoma, FSCN-1 in esophageal squamous cell carcinoma, N-cadherin in gastric carcinoma, and IGF in hepatocellular carcinoma [38–41]. In our experiments, miR-145 expression in the urothelial carcinoma cell lines T24 and KU7 induces cell senescence without apoptosis. We have previously shown syndecan-1 to be important for the tumorigenicity and serial reproducibility of prostatic tumor-initiating cells, as well as for forming tumorigenic microspheres [12]. Indeed, syndecan-1 might contribute to cell survival and progression, and is up-regulated in urothelial carcinomas, particularly in high-grade and aggressively invasive cancers [19]. Together with these observations, miR-145 provides the basis for differentiation into multiple cell types, and for suppression of tumor-initiating cells.

Approximately 80 % of urothelial carcinomas are low-grade and non-invasive, and about 70 % are multifocal or recurrent with reasonably good prognosis [21]. In contrast, high-grade and invasive cancers metastasize locally and distally. The capacity of such cancers to invade other tissues depends on intrinsic genetic factors, such as mutations in Ras or fibroblast growth factor receptor 3, as well as expression of p53 and RB tumor suppressor [42]. Reduced E-cadherin expression compromises cell-cell adhesion, and is histologically associated with high-grade, invasive urothelial carcinoma [21]. However, our data show that miR-145 overexpression induces E-cadherin, but not p53, and that miR-145 is more abundantly expressed in low-grade urothelial carcinomas than in high-grade cancers. Therefore, miR-145 may additionally suppress tumors by enhancing cell-cell adhesion through E-cadherin. Notably, E-cadherin expression is negatively correlated with syndecan-1 expression, which is in line with previous results [19].

## Conclusion

In conclusion, miR-145 utilizes syndecan-1 to modulate cell proliferation, re-programming, and differentiation in urothelial carcinomas. Thus, miR-145 is a potential diagnostic or prognostic marker, as well as a target for therapy.

## Additional file

Additional file 1: Figure S1. mRNA expression of differentiation markers, stem cell markers and syndecan-1 in KU7 cells. Y-axis in A, B, C and D was indicative of relative mRNA expression compared with control. All mRNAs data were normalized by GAPDH. (A) mRNA expression of squamous markers (p63 and TP63 ; left graph), glandular markers (MUC-1, MUC-2 and MUC-5 AC ; center graph) and neuroendocrine markers (NSE and USHL-1 ; right graph) was increased under conditions of miR-145 overexpression in KU7 cells. (B) mRNA expression of stem cell markers (SOX2, NANOG, OCT4, and E2F3) was increased, but not CD44 under conditions of overexpression of miR-145 in KU7 cells. (C) E-cadherin expression was increased, but not p53 under transfection of miR-145 precursor. (D) Syndecan-1 mRNA expression was suppressed by transfection of miR-145 precursor. (**p* < 0.05, miR-145 pre; miR-145 precursor). (JPEG 382 kb)
