# Supplementary figures and images for: microRNA-145 promotes differentiation in human urothelial carcinoma through down-regulation of syndecan-1
Source: BMC Cancer. 2015 Oct 29;15:818. doi: 10.1186/s12885-015-1846-0 (PMC4625524; doi:10.1186/s12885-015-1846-0)

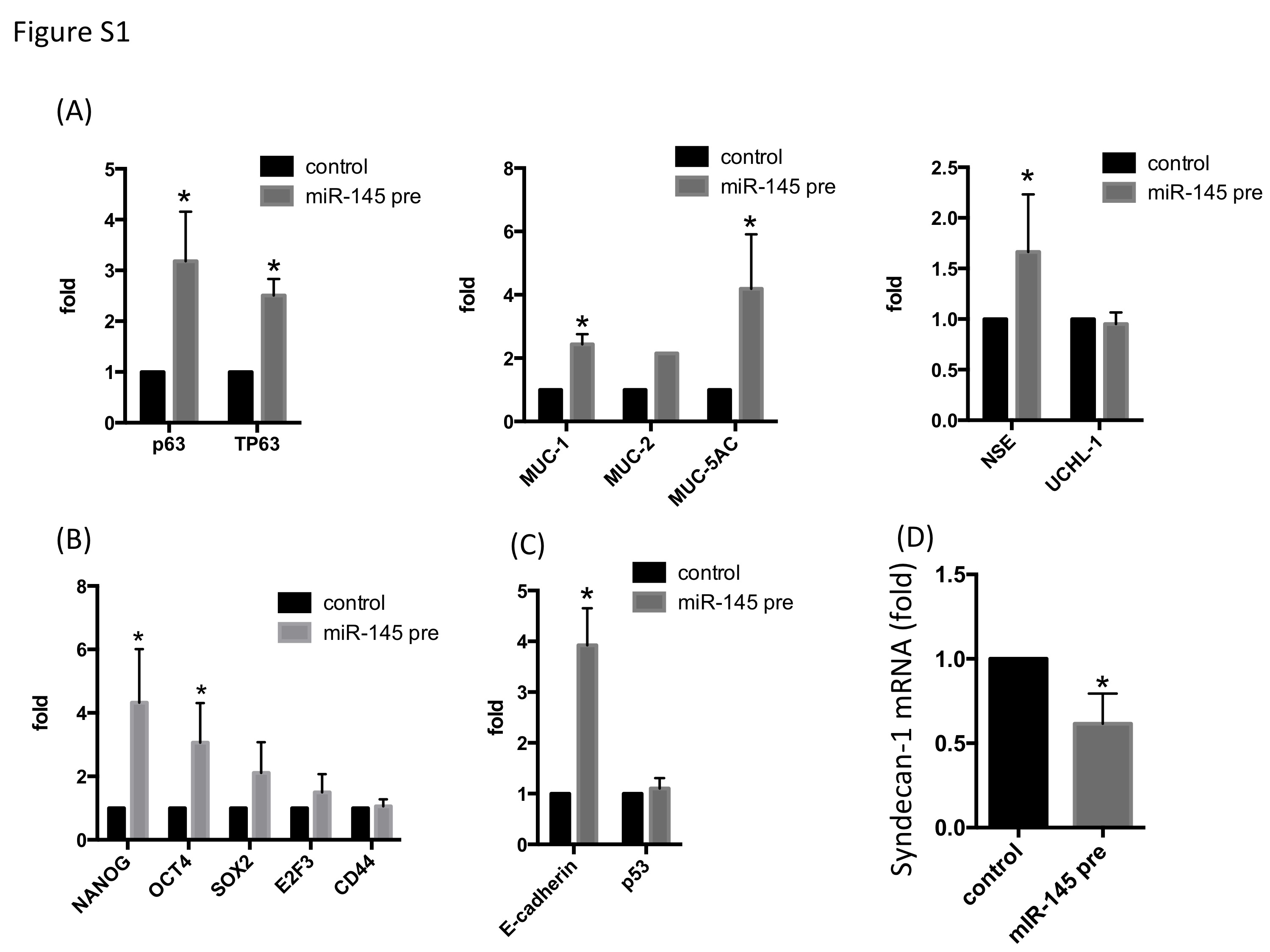

Supplement: Additional file 1: Figure S1. — mRNA expression of differentiation markers, stem cell markers and syndecan-1 in KU7 cells. Y-axis in A, B, C and D was indicative of relative mRNA expression compared with control. All mRNAs data were normalized by GAPDH. (A) mRNA expression of squamous markers (p63 and TP63 ; left graph), glandular markers (MUC-1, MUC-2 and MUC-5 AC ; center graph) and neuroendocrine markers (NSE and USHL-1 ; right graph) was increased under conditions of miR-145 overexpression in KU7 cells. (B) mRNA expression of stem cell markers (SOX2, NANOG, OCT4, and E2F3) was increased, but not CD44 under conditions of overexpression of miR-145 in KU7 cells. (C) E-cadherin expression was increased, but not p53 under transfection of miR-145 precursor. (D) Syndecan-1 mRNA expression was suppressed by transfection of miR-145 precursor. (*p < 0.05, miR-145 pre; miR-145 precursor). (JPEG 382 kb) [file 12885_2015_1846_MOESM1_ESM.jpeg]
